# A tagging SNP in *INSIG2 *is associated with obesity-related phenotypes among Samoans

**DOI:** 10.1186/1471-2350-10-143

**Published:** 2009-12-22

**Authors:** Ranjan Deka, Ling Xu, Prodipto Pal, Palanitina T Toelupe, Tuiasina S Laumoli, Huifeng Xi, Ge Zhang, Daniel E Weeks, Stephen T McGarvey

**Affiliations:** 1 Center for Genome Information, Department of Environmental Health, University of Cincinnati College of Medicine, 3223 Eden Avenue, Cincinnati, OH 45267, USA; 2 Ministry of Health, Government of Samoa, Apia, Samoa; 3 Department of Health, American Samoa Government, Pago Pago, American Samoa; 4 Departments of Family Medicine and Environmental Health, University of Cincinnati College of Medicine, 3223 Eden Avenue, Cincinnati, OH 45267, USA; 5 Departments of Human Genetics and Biostatistics, Graduate School of Public Health, University of Pittsburgh, 130 DeSoto St, Pittsburgh, PA 15261, USA; 6 International Health Institute, Brown University, 121 S Main St, Providence, RI 02912, USA

## Abstract

**Background:**

A genome wide association study found significant association of a sequence variant, rs7566605, in the insulin-induced gene 2 (*INSIG2*) with obesity. However, the association remained inconclusive in follow-up studies. We tested for association of four tagging SNPs (tagSNPs) including this variant with body mass index (BMI) and abdominal circumference (ABDCIR) in the Samoans of the Western Pacific, a population with high levels of obesity.

**Methods:**

We studied 907 adult Samoan participants from a longitudinal study of adiposity and cardiovascular disease risk in two polities, American Samoa and Samoa. Four tagSNPs were identified from the Chinese HapMap database based on pairwise *r*^*2 *^of ≥0.8 and minor allele frequency of ≥0.05. Genotyping was performed using the TaqMan assay. Tests of association with BMI and ABDCIR were performed under the additive model.

**Results:**

We did not find association of rs7566605 with either BMI or ABDCIR in any group of the Samoans. However, the most distally located tagSNPs in Intron 3 of the gene, rs9308762, showed significant association with both BMI (p-value 0.024) and ABDCIR (p-value 0.009) in the combined sample and with BMI (p-value 0.038) in the sample from Samoa.

**Conclusion:**

Although rs7566605 was not significantly associated with obesity in our study population, we can not rule out the involvement of *INSIG2 *in obesity related traits as we found significant association of another tagSNP in *INSIG2 *with both BMI and ABDCIR. This study suggests the importance of comprehensive assessment of sequence variants within a gene in association studies.

## Background

Using genome-wide association analysis, Herbert et al. [1] reported a common variant, rs7566605, in the 5' region of the *INSIG2 *gene associated with obesity in the Framingham Heart Study population samples and also replicated this finding in four independent cohorts of European and African American ancestries. However, the association of this variant remains inconclusive with confirmation or lack thereof in several follow-up studies conducted in populations of diverse ethnicities [2-12]. Analysis of a single variant could be perceived as a limitation in assessing genetic association of a putative locus. To guard against this potential limitation we conducted a comprehensive association analysis of common tagging SNPs in the *INSIG2 *gene among adult Samoans, Polynesians of the Western Pacific, residing in Samoa and American Samoa.

## Methods

Our study sample derives from a longitudinal study of adiposity and cardiovascular disease risk factors performed in American Samoa and Samoa from 1990 to 1995. Compared to most worldwide populations, the levels of overweight and obesity are remarkably high among Samoans [13-15]. Our sample of Samoans was collected in two territories, the independent nation of Samoa and the U.S. territory of American Samoa. Although there is substantial economic disparity between the two polities, Samoans from both territories form a single socio-cultural unit with frequent exchange of mates and genetically they represent a single homogenous population without any evidence for significant population substructure as supported by our prior genetic analyses [16-18]. Participants were between 18 and 84 years of age with all four grandparents of Samoan ancestry. Detailed descriptions of the sampling and recruitment are reported previously [19,20]. Anthropometric measurements at the baseline in 1990 and 1991 were obtained following standard protocols. For this study, two phenotypes of interest, used as surrogates for obesity, were BMI ((weight in kg)/(height in m^2^)) and ABDCIR (in cm). This study sample was restricted to those 907 participants with a fasting glucose level of ≤110 mg/dL in order to reduce the confounding of hyperglycemia and type 2 diabetes on the obesity traits. Two of the 907 subjects were on anti-diabetic medication. The mean age was 38.5 years (38.1 in Samoa and 39.0 in American Samoa). The mean BMI and ABDCIR were higher in American Samoa compared to Samoa (Table 1). Study protocols were approved by the Institutional Review Boards of the Miriam Hospital and Brown University, Providence, RI, and officials of the Samoa Ministry of Health and the Department of Health in American Samoa. Written informed consent was obtained from all participants.

**Table 1:** Summary of participants, age and basic statistics (Mean ± SD) of the two phenotypes

	Age (yrs)	BMI (kg/height in m^**2**^) Range: 17.9 - 62.3	ABDCIR (cm) Range: 65.6 - 164.1
**Combined sample (N = 907)**	38.5 ± 9.3	32.0 ± 6.1	100.4 ± 15.1
Male (N = 404)	38.7 ± 9.3	30.9 ± 5.8	98.0 ± 14.8
Female (N = 503)	38.3 ± 9.3	32.9 ± 6.2	102.4 ± 15.1
**Samoa (N = 502)**	38.1 ± 8.7	30.0 ± 5.0	95.3 ± 13.1
Male (N = 235)	38.2 ± 8.7	29.1 ± 4.8	92.8 ± 12.5
Female (N = 267)	38.0 ± 8.7	30.9 ± 5.1	97.4 ± 13.3
**American Samoa (N = 405)**	39.0 ± 10.0	34.4 ± 6.3	106.7 ± 15.0
Male (N = 169)	39.5 ± 10.0	33.3 ± 6.0	105.1 ± 14.9
Female (N = 236)	38.7 ± 9.9	35.2 ± 6.5	107.9 ± 15.0

**Figure 1:**
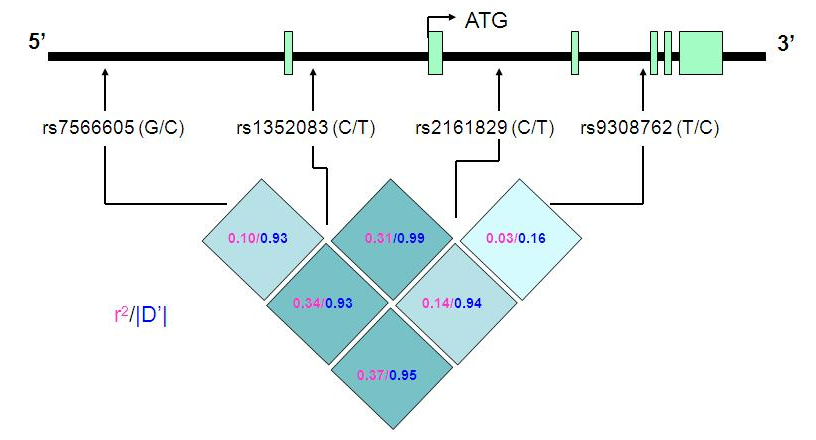
**Schematic representation of the *INSIG2 *gene region showing the relative location of the tagging SNPs from 5' end to the 3' end of the gene**. Allelic variants at each SNP are shown in parenthesis following the 'rs' numbers of the SNPs; the second allele at each locus represents the minor allele. Linkage disequilibria values, r^2 ^and D', among the SNPs are shown in the boxes.

Tagging SNPs were selected based on pairwise *r*^*2 *^(≥0.8) among all common SNPs with minor allele frequency (MAF) ≥0.05 using the approach of Carlson et al. [21]. We used the Chinese HapMap database http://www.hapmap.org. Previously we had shown the portability of the tagging SNPs and LD sharing between the Chinese and the Samoans [22]. Four tagSNPs spanning a region of 28 kb in *INSIG2 *including rs7566605 were identified (Figure 1). Genotyping was performed using the TaqMan assay (Applied Biosystems).

We used the GenABEL package [23] in R to test for association of the SNPs under the additive model. Empirical p-values maximized over the four SNPs were computed using the emp.qtscore function with 10,000 replicates. Since these empirical p-values adjust for the four SNPs, for each trait, we have carried out 5 tests on various subdivisions of the data. A stringent multiple testing correction for this would be to use the Bonferroni correction, which would require testing at the 0.05/5 = 0.01 level. We adjusted for age in all samples, for location (Samoa and American Samoa) in the combined samples and for sex in the Samoa and American Samoa samples. Of the two obesity measurements, BMI was not normally distributed; therefore, we performed logarithmic transformation of BMI to obtain normality.

## Results and Discussion

**Figure 2:**
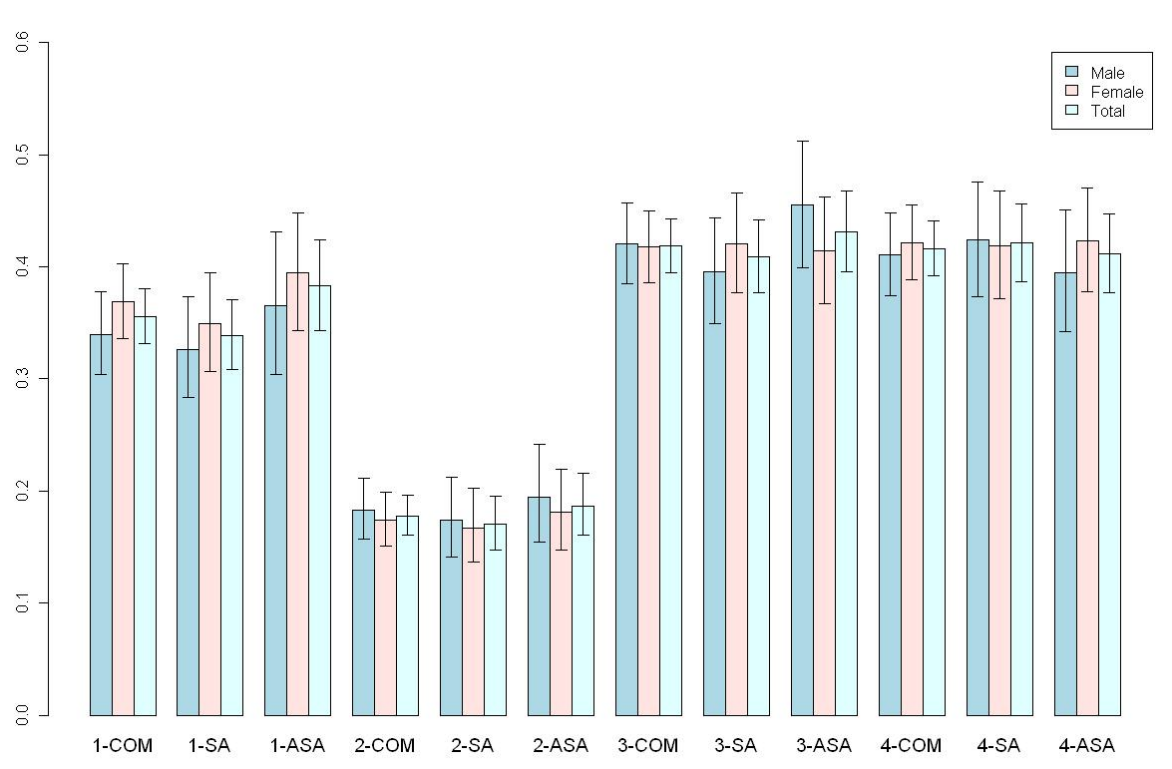
**Diagrammatic representation of the distribution of minor allele frequencies with 95% confidence intervals at each SNP in the combined (COM), Samoa (SA), and America Samoa (ASA) samples**. Preceding each population designations are the sequential SNP numbers from the 5' to the 3' end, e.g., 1-COM denotes rs7566605 in the combined sample.

All of the four SNPs are polymorphic in both locations with minor allele frequencies (MAF) ranging from 17% to 42% (Figure 2). All four SNPs are in HWE (data not shown) with the exception of marginal departures at rs1352083 in the combined male sample (p = 0.053) and rs2161829 in the Samoa female sample (p = 0.051). Since we did not observe significant allele frequency difference between the two polities, we combined the samples and performed tests of association based on the additive model (Table 2). Males and females in this combined sample did not show association of any of the four tagging SNPs either with BMI or ABDCIR. However, when males and females were combined, rs9308762 showed significant association with both BMI (p = 0.024) and ABDCIR (p = 0.009). These results are similar when the test statistic was performed under the recessive model, with both BMI (p = 0.038) and ABDCIR (p = 0.018) showing association with rs9308762. Note that the initial study used the recessive model to fit the association signal [1]. Under additive model, each copy of C allele of rs9308762 was associated with an increase of age and gender adjusted BMI = 0.775 kg/m^2 ^and ABDCIR = 2.08 cm. Then we performed tests of associations separately for samples from Samoa and American Samoa. The results showed a significant association of rs9308762 with BMI in the sample from Samoa, but not in the American Samoa sample.

**Table 2:** Association (empirical p-values) of *INSIG2 *tagSNPs with BMI and ABDCIR based on additive model (adjusted for age in all samples, for location in the combined samples and for sex in the Samoa and American Samoa samples).

	rs7566605		rs1352083		rs2161829		rs9308762	
	
	BMI	ABDCIR	BMI	ABDCIR	BMI	ABDCIR	BMI	ABDCIR
Combined Male	0.359	0.223	0.690	0.629	0.659	0.217	0.386	0.128
Combined Female	0.988	1.000	0.587	0.263	1.000	0.995	0.063	0.076
Combined Total	0.498	0.563	0.999	0.973	0.898	0.423	**0.024**	**0.009**
Samoa	0.411	0.547	0.999	0.999	0.955	0.642	**0.038**	0.070
Am Samoa	0.998	0.991	1.000	0.959	0.994	0.854	0.618	0.236

Although we did not find association of rs7566605 with either BMI or ABDCIR, the *INSIG2 *gene itself is of considerable interest for obesity-related quantitative traits as it is implicated in lipid regulation and synthesis [24,25]. It should be noted that rs7566605 is located 10 kb upstream of *INSIG2 *and its functional relevance is not yet known. The putative association of rs7566605 could have resulted from linkage disequilibrium (LD) with a functional variant in *INSIG2 *in the study populations, in which the association was detected. The follow-up replication studies considered only this polymorphism with inconsistent results in populations irrespective of their ethnic affiliations [2-12,26]. Among the Asian populations with whom the Samoans share a more recent ancestry, the association of rs7566605 with obesity has been largely negative [27-30], although one study found significant association with BMI in a population from Uyghur [31]. On account of genetic diversity across populations, the extent of LD among the genetic variants is likely to vary, and this could be one of the reasons for the inconsistent findings. To guard against this potential limitation, we conducted an association analysis of common tagging SNPs in *INSIG2 *with two obesity-related phenotypes. It should be noted that using both genome-wide microsatellite markers at a spacing of 10 cM and over 7,000 SNPs distributed on chromosome 21, we observed a significantly higher level of LD in the Samoans compared to the European populations [18,22] favoring the identification of a surrogate marker further away from the true variant in the relatively isolated Samoan population. We observed significant association of the most distal SNP rs9308762 with both BMI and ABDCIR in the combined Samoan sample. Although this observation is not in complete agreement with the previous report in which the most significant association was found with rs7566605 [1], our results do not necessarily indicate the existence of different functional variants. A possible explanation is that there is a single functional variant, which is in LD with both rs7566605 and rs9308762. Note that these two SNPs are in elevated LD in the Samoan as well as in the HapMap Chinese population, r^2 ^= 0.37 and 0.45, respectively. It is plausible that the putative causal variant is more strongly associated in Europeans with rs7566605, while in Samoans the stronger association is with rs9308762. Although the observations are somewhat preliminary, these results reaffirm the involvement of *INSIG2 *in regulation of body weight or pathophysiology of obesity related phenotypes.

## Conclusion

Although rs7566605 was not significantly associated with obesity in the Samoan population, this study suggests that sequence variants in *INSIG2 *likely influence the risk for obesity related traits. Our study reiterates the importance of comprehensive assessment of genetic variants within a gene in association studies.

## Competing interests

The authors declare that they have no competing interests.

## Authors' contributions

STM, DEW and RD collaboratively contributed to the conception and design of the study and drafted the manuscript. PP and HX conducted genotyping of all samples. LX and GZ performed statistical analysis. PTT and TSL provided support and advice for the field work in Samoa and American Samoa, respectively. All authors contributed to the writing of the manuscript, read and approved the final version.

## Pre-publication history

The pre-publication history for this paper can be accessed here:

http://www.biomedcentral.com/1471-2350/10/143/prepub
